# Systemic sclerosis associated with silicosis in an agricultural worker: A case of Erasmus syndrome

**DOI:** 10.1016/j.radcr.2026.02.054

**Published:** 2026-03-23

**Authors:** Saleck Choumad, Hind Qajia, Ahmed Ebedda, Aichetou Mohamed El Hacen, Youssef Omor, Fatima zahra Laamrani, Rachida Latib, Sanae Amalik

**Affiliations:** a Department of Radiology, National Institute of Oncology, UHC Ibn Sina Mohammed V University, Rabat, Morocco; b Department of Central Radiology, National Institute of Oncology, UHC Ibn Sina Mohammed V University, Rabat, Morocco

**Keywords:** Systemic sclerosis, Silicosis, Thoracic imaging, Autoimmunity, Anamnesis

## Abstract

Erasmus syndrome is a rare entity defined by the association of systemic sclerosis and documented silica exposure, with or without established silicosis. A thoraco-abdominopelvic CT scan, performed during surveillance of treated ovarian carcinoma in a 59-year-old woman, revealed multiple calcified micronodules, mediastinal and hilar lymph node calcifications with an “egg-shell” pattern, traction bronchiectasis, and esophageal dilatation, suggesting silicosis. A directed history disclosed more than 30 years of non-industrial agricultural exposure to silica-rich dust during manual processing of lentils and beans in a desert region. Serologic testing demonstrated high-titer anti-Scl-70 antibodies, confirming systemic sclerosis and supporting the diagnosis of Erasmus syndrome. This case highlights the pivotal role of thoracic CT in detecting characteristic silicotic patterns and illustrates how imaging can prompt targeted occupational anamnesis in non-industrial exposure settings.

## Introduction

Erasmus syndrome is a rare disorder characterized by the coexistence of systemic sclerosis and documented exposure to silica, with or without radiologic evidence of silicosis [[1], [2], [3]]. First described in South African gold miners, it represents a paradigm of environmentally triggered autoimmune disease [4].

Although classically associated with mining, stone cutting, or sandblasting, similar cases have been reported in agricultural workers exposed to silica-rich soil dust [5,6].

In many patients, thoracic CT provides the first diagnostic clue, revealing characteristic silicotic patterns that prompt targeted occupational anamnesis and appropriate serologic testing, allowing earlier recognition of Erasmus syndrome.

## Case report

A 59-year-old woman with a history of treated ovarian carcinoma underwent routine thoraco-abdominopelvic CT for oncologic surveillance.

CT demonstrated bilateral multiple calcified pulmonary micronodules with centrilobular and perilymphatic distribution, predominantly in the upper and middle lung zones, associated with bilateral mediastinal and hilar “egg-shell” lymph node calcifications, traction bronchiectasis, and diffuse esophageal dilatation. No evidence of recurrent malignancy was identified.

High-resolution CT reconstructions were available, confirming micronodular distribution and interstitial involvement. No features of progressive massive fibrosis were observed.

Directed anamnesis revealed more than 30 years of agricultural work in a desert region with chronic exposure to silica-laden dust during manual threshing of lentils and beans.

Clinically, the patient reported progressive exertional dyspnea over 2 years, diffuse arthralgias, Raynaud’s phenomenon, and gradual cutaneous thickening of the hands and face.

Pulmonary function tests showed a mild restrictive pattern with reduced DLCO (insert exact values if available).

Serology revealed positive ANA and high-titer anti-Scl-70 antibodies, fulfilling the 2013 ACR/EULAR classification criteria for systemic sclerosis.

**Fig. 1:**
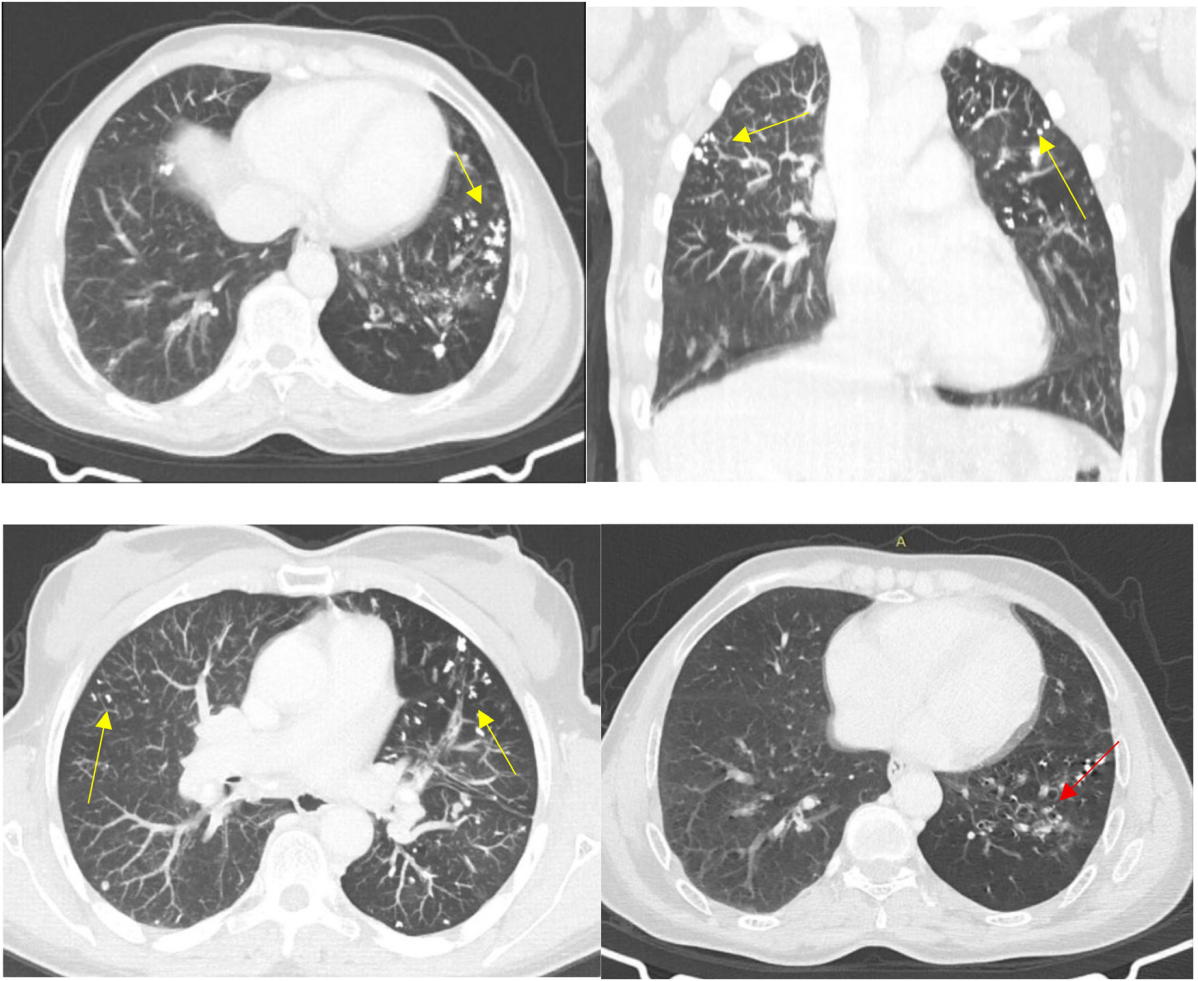
Axial and coronal CT images (lung window) demonstrating multiple predominantly upper-lobe calcified micronodules (yellow arrow) with centrilobular and perilymphatic distribution, associated with traction bronchiectasis (red arrow).

**Fig. 2:**
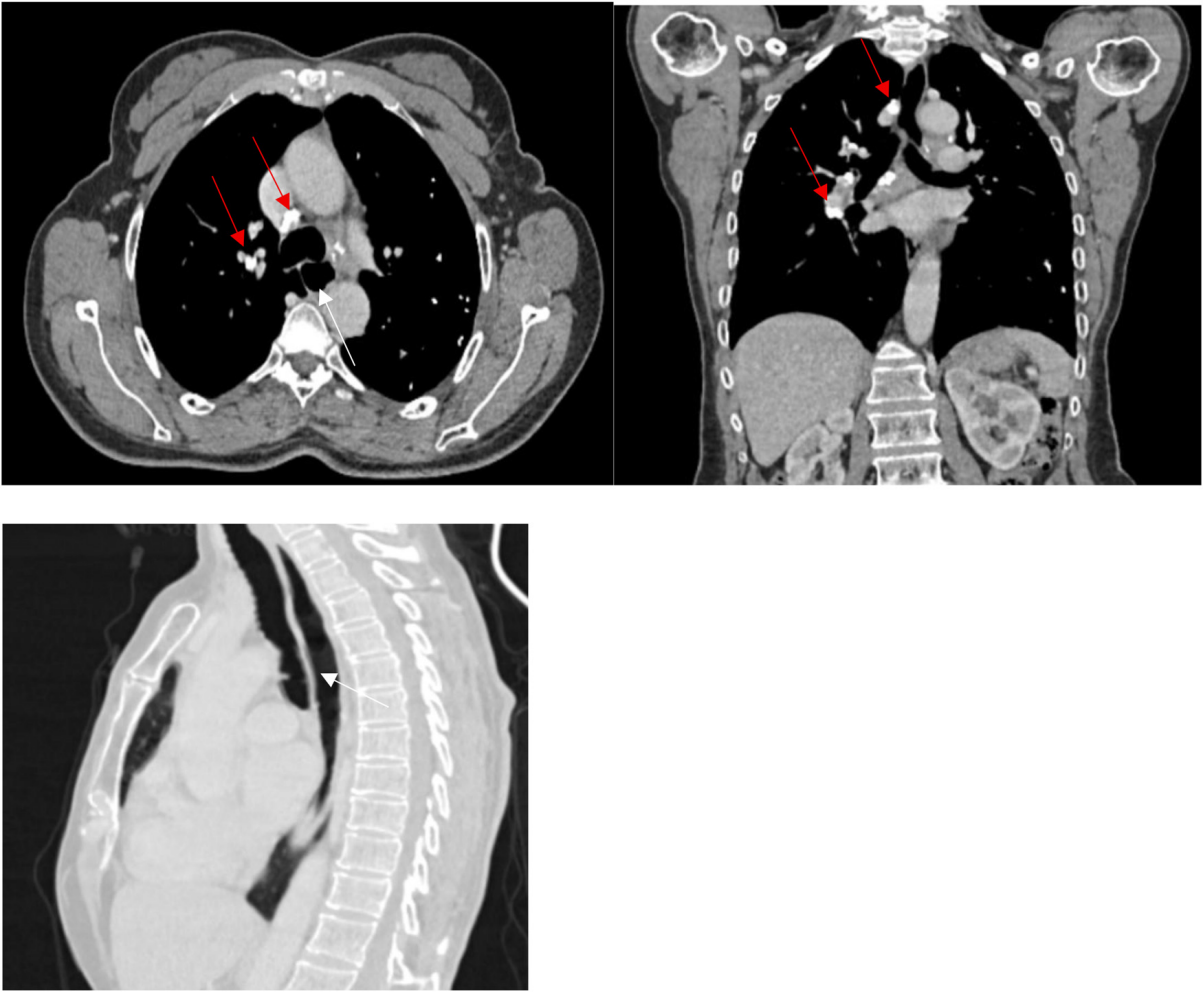
Axial and coronal CT images (mediastinal window) and sagittal CT image (parenchymal window) demonstrating mediastinal and hilar lymph node calcifications (red arrow) and esophageal dilation (white arrow).

The combination of characteristic CT findings, documented silica exposure, and autoimmune serology led to the diagnosis of Erasmus syndrome. Treatment with mycophenolate mofetil and nifedipine was initiated, and the patient was advised to avoid further silica exposure (Fig. 1, Fig. 2).

## Discussion

Erasmus syndrome represents the intersection between silica exposure and autoimmune fibrosing disease, namely, systemic sclerosis [1,7].

It is defined by the occurrence of systemic sclerosis in individuals exposed to silica, whether or not radiologic silicosis is evident.

Silica inhalation triggers macrophage activation and the release of proinflammatory and profibrotic cytokines (IL-1, TNF-α, and TGF-β), resulting in fibrosis and autoimmune dysregulation in genetically susceptible individuals [[8], [9], [10]].

CT imaging, especially high-resolution CT, is pivotal in diagnosing Erasmus syndrome [9,10].

It allows detection of silicotic nodules, characteristic egg-shell lymph node calcifications, and esophageal involvement related to systemic sclerosis.

In many cases, imaging prompts the clinician to pursue a targeted environmental history, leading to diagnosis before the disease is clinically advanced.

In this case, thoracic CT showed calcified micronodules and egg-shell lymph node calcifications, highly suggestive of silicosis. Given the oncologic history, several differential diagnoses were considered.

### Calcified pulmonary metastases

In the context of prior ovarian cancer, calcified metastases were considered. However, metastatic nodules are typically random, variable in size, and irregular in contour, lacking the uniform 2-5 mm micronodules and characteristic egg-shell lymphadenopathy seen in silicosis. The stability of findings and absence of new lesions further supported a non-neoplastic etiology.

### Sarcoidosis

While sarcoidosis may show perilymphatic micronodules and bilateral hilar lymphadenopathy, the presence of densely calcified micronodules with classic egg-shell lymph node calcifications in a patient with documented silica exposure strongly favors silicosis over sarcoidosis [11]. Moreover, sarcoidosis rarely demonstrates the same pattern of calcification or associated upper-lobe fibrosis with traction bronchiectasis.

Sarcoid-related calcifications are usually central or punctate, and systemic involvement (cutaneous, ocular) is frequently present, which was not the case here.

### Post-tuberculous sequelae

Healed tuberculosis may cause nodal and parenchymal calcifications, but these are usually irregular, coarse, and central, often associated with fibrotic retraction or cavitations [12].

The absence of cavities, regular peripheral rim calcifications, and autoimmune antibody positivity excluded this diagnosis.

### Thoracic amyloidosis

Nodular pulmonary amyloidosis can mimic silicosis with calcified nodules, but these tend to be few, large, and non-systematic and are not accompanied by egg-shell lymphadenopathy [13].

The diffuse, micronodular pattern and lack of systemic amyloid features favored silicosis.

This case underscores the critical role of thoracic radiologists in recognizing characteristic silicotic patterns, particularly in patients without classic industrial exposure. In this patient, CT findings prompted a targeted occupational history that ultimately led to the diagnosis of Erasmus syndrome, demonstrating how imaging can initiate the diagnostic pathway in autoimmune lung disease (Table 1).

**Table 1:** Differential diagnosis of calcified pulmonary micronodules and lymph node calcifications on CT.

Feature	Silicosis/Erasmus syndrome	Calcified pulmonary metastases	Sarcoidosis	Post-tuberculous sequelae	Thoracic amyloidosis
Clinical context	Chronic silica exposure (including agriculture); possible systemic sclerosis	Known malignancy (osteosarcoma, chondrosarcoma, mucinous carcinoma)	Multisystem granulomatous disease	Previous tuberculosis	Localized or systemic amyloidosis
Nodule distribution	Centrilobular and perilymphatic, upper-lobe predominance	Random or basal	Perilymphatic, mid- to upper-lung zones	Apical/posterior segments	Random or subpleural
Nodule morphology	Multiple small (2-5 mm), densely calcified, uniform	Variable size, irregular or spiculated, heterogeneous calcification	Usually non-calcified or punctate calcifications	Coarse, irregular central calcifications	Few, larger nodules with amorphous calcification
Lymph node calcification	Classic “egg-shell” peripheral rim	Rare or amorphous	Central or punctate	Irregular, coarse central	Usually absent
Associated parenchymal findings	Upper-lobe fibrosis, traction bronchiectasis; possible emphysematous changes	Possible pleural nodules or growth on follow-up	Bilateral hilar adenopathy; extrapulmonary involvement	Fibrotic bands, volume loss, cavities	Tracheobronchial plaques or masses
Behavior on follow-up CT	Generally stable or slowly progressive	Often progressive	Variable	Usually stable	Variable
Immunologic/clinical clues	Positive autoimmune markers (eg anti-Scl-70)	Tumor markers/oncologic history	Elevated ACE; noncaseating granulomas	TB history or scars	Congo-red positivity

## Conclusion

Erasmus syndrome is a rare autoimmune complication of chronic silica exposure, combining silicosis with systemic sclerosis. The presence of calcified micronodules and egg-shell mediastinal lymphadenopathy is highly suggestive of silicosis, even in non-industrial settings.

## Key imaging takeaway for thoracic radiologists

When characteristic silicotic patterns are identified on CT—particularly egg-shell lymphadenopathy—targeted occupational anamnesis and autoimmune testing should be initiated, as these findings may reveal underlying systemic sclerosis consistent with Erasmus syndrome.

## Authors' contributions

All authors contributed to the implementation and realization of this work. All authors declare that they have read and approved the final version of this manuscript.

## Patient consent

Written informed consent was obtained from the patient(s) for their anonymized information to be published in this article.
